# Developing and characterizing a two-layered safety switch for cell therapies

**DOI:** 10.1080/15384047.2023.2232146

**Published:** 2023-07-13

**Authors:** Filippo Rossignoli, Danielle Hoffman, Emaan Atif, Khalid Shah

**Affiliations:** a Center for Stem Cell and Translational Immunotherapy (CSTI), Harvard Medical School, Boston, MA, USA; b Department of Neurosurgery, Brigham and Women’s Hospital, Boston, MA, USA; c Harvard Stem Cell Institute, Harvard University, Boston, MA, USA

**Keywords:** safety switch, suicide genes, cell therapy, clinical translation, adverse reaction

## Abstract

Gene edited and engineered cell-based therapies are a promising approach for treating a variety of disorders, including cancer. However, the ability of engineered cells to persist for prolonged periods along with possible toxicity raises concerns over the safety of these approaches. Although a number of different one-dimensional suicide systems have been incorporated into therapeutic cell types, the incorporation of a two-layered suicide system that allows controlled killing of therapeutic cells at different time points is needed. In this study, we engineered a variety of therapeutic cells to express two different kill switches, RapaCasp9 and HSV-TK and utilized Rapamycin and Ganciclovir respectively to activate these kill switches. We show that the function of both RapaCasp9 and HSV-TK molecules is preserved and can be activated to induce apoptosis detected early (24 h) and late (48 h) post-activation respectively, with no toxicity. In vivo, we show the eradication of a majority of cells after treatment in subcutaneous and orthotopic models. Furthermore, we demonstrate how both suicide switches work independently and can be activated sequentially for an improved killing, thus ensuring a failsafe mechanism in case the activation of a single one of them is not sufficient to eliminate the cells. Our findings highlight the reliability of the double suicide system, effective on a variety of cells with different biological characteristics, independent of their anatomic presence.

## Introduction

In recent years, engineered cells have been at the forefront of advanced therapies to address a wide variety of disorders, and, in particular, for treating various forms of cancer. Strategies such as Adoptive Cellular Therapies (ACT), which comprise administering immune cells to cancer patients to mediate anti-tumor function, have become increasingly popular, particularly in the clinical setting^1^. Moreover, several other pre-clinical and clinical therapeutic strategies to treat various neoplastic and non-neoplastic conditions involve the administration of cellular products such as stem cells^2^, mesenchymal stem cells (MSC)^3^, iPSC-derived cells^4^, immortalized cell lines^5^, and even engineered tumor cells^6^. In all these cases, the possibility of an unpredictable behavior of the cells post-treatment necessitates the development and use of a system to control their fate efficiently in the long term. In fact, the clinical trials often uncover severe adverse events which limit the reliability of these approaches^1,7^. In detail, “on target-off tumor” toxicity is observed when a response is directed against healthy tissues due to a cross-recognition of the target antigen^1,7^. Another life-threatening condition, the cytokine release syndrome, is an “on target-on tumor” adverse event, involving a massive cytokine release triggered by the infused cells leading to an uncontrolled immune activation^1,7^. Finally, other “off target-off tumor” severe conditions observed after ACT include IgE-mediated anaphylactic reactions and neurological complications of uncertain etiology^1,7^.

**Figure 1.**
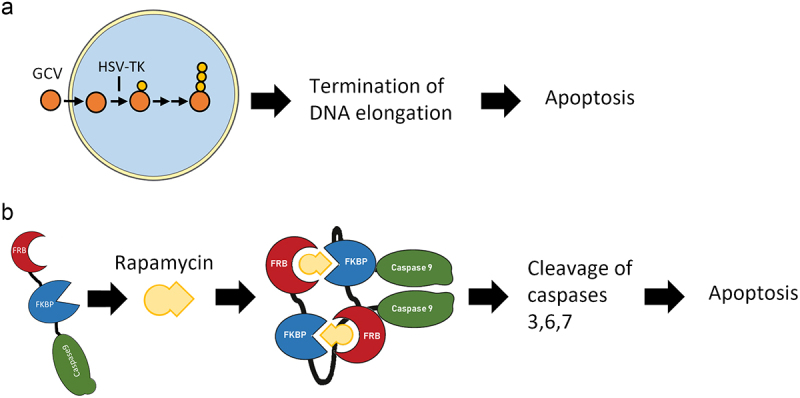
Schematic representation of the double kill switch components mechanism of action. The double suicide kill switch integrates the functions of RapaCasp9 and HSV-TK suicide systems. (a) RapaCasp9 molecule is composed by the fusion of an FRB and an FKBP domains to the catalytic domain of Caspase9. Rapamycin molecule causes the homo-dimerization of RapaCasp9 with the consequent activation of the Caspase9 domain and the cleavage of executioner Caspases 3, 6, and 7 that lead the cell to apoptosis. (b) HSV-TK molecule catalyses the phosphorylation of ganciclovir in the cell cytoplasm. The intracellular kinases are then responsible for its further conversion into a tri-phosphorylated form. The molecule competes with deoxyguanosine incorporation during DNA elongation, resulting in cell death.

To make these treatments safer and, in particular, to control unexpected toxicities, uncontrolled cell expansion and managing the substantial number of infused therapeutic cells, the introduction of inducible suicide genes has proven to be beneficial^8,9^. A well-known class of these systems involves genes, such as Herpes Simplex Virus Thymidine Kinase (HSV-TK) and Cytosine Deaminase, which products acts by converting a typically nontoxic prodrug into a cytotoxic drug eventually leading the cell to death^10^. HSV-TK, in particular, phosphorylates the ordinarily inert Ganciclovir (GCV) which is then further modified by the host cell, thus obtaining a highly toxic compound which induces cell apoptosis (Figure 1a)^11^. Another class of suicide genes is based on engineered forms of Caspase 9 in which dimerization and consequent activation relies on the presence of a small-molecule inducer of dimerization^12^. Among these, a relatively new system is the Rapamycin-activated Caspase9 system (RapaCasp9). RapaCasp9 consists of an FRB and FKBP domain fused to the catalytic domain of caspase 9. Rapamycin, a marketed immune-suppressive drug, can bind to the FRB domain of one molecule and simultaneously recruit the FKBP domain of another, thus dimerizing and activating the caspase 9 domain, leading the cell into apoptosis (Figure 1b)^13^. A big advantage of this system is that Rapamycin is a small molecule, making it a valid option for peculiar sites such as the blood-brain barrier or poorly vascularized tissues^13^.

While the use of suicide genes as safety switches has shown favorable results in eliminating aberrant cell populations, studies have also shown that some cells evade the elimination and can regrow and persist for longer periods^14,15^. In particular, Zhou et al. report how, in a long-term follow-up of 10 patients infused with iCasp9-expressing T cells, the activation of the safety switch was unable to fully eradicate T cells. These cells remained detectable for months and even re-expanded in a subset of patients^15^. Other studies have shown that multiple doses of induction lead to a progressively lower percentage of killing, with the potential of therapeutic cells becoming resistant to the pro-drug in the long-term, thus escaping eradication^16^. In this study, we explored the incorporation of two-layered suicide gene systems of HSV-TK/GCV and RapaCasp9/Rapamycin in several therapy-relevant cell types in vitro and in vivo. In the cases where one suicide switch is not enough to efficiently control the persistence of engineered cells, a second one can act as a backup and eliminate any more remaining. Our models confirm the robustness, reliability, and broad applicability of the strategy, showing its potential to improve the safety of cell therapies.

## Results

### RapaCasp9 and HSV-TK successfully eliminate transduced cells in vitro as single agents

**Figure 2.**
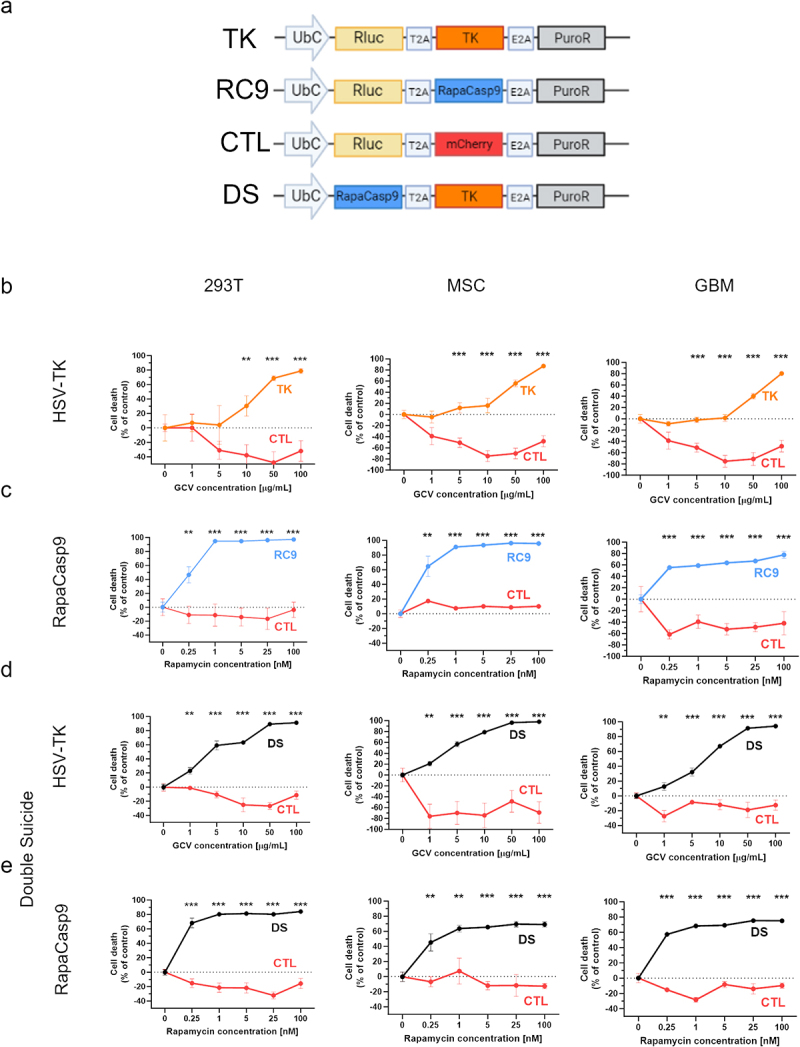
The double kill switch efficiently eradicates transduced cells in vitro. (a) Schematic representation of the expression cassettes used. The two suicide genes Thymidine Kinase (TK) and RapaCasp9 (RC9) are incorporated in a cassette expressing renilla luciferase (Rluc) and Puromycin resistance (PuroR) genes. In the double suicide (DS) plasmid, RapaCasp9 and TK genes are expressed together. The control plasmid uses mCherry molecule in place of the kill switch. (b) the activation of HSV-TK kill switch by incubation with GCV eliminates 293T, MSC and GCV cells in a dose-dependent fashion. (c) incubation of transduced cells with Rapamycin, induces their death starting at a very low dose. (d and e) cells bearing the DS gene, are sensitive to both activation of TK gene by GCV and activation of RapaCasp9 gene by Rapamycin. Data for each experimental condition, have been collected from two independent experiments in technical triplicate. Cell death between different groups has been tested for significance for every concentration using student’s T Test. *p < .05, **p < .01, ***p < .001.

293T cells, MSC and tumor cells (TC) were transduced with lentiviral particles bearing RapaCasp9 (293T-RC9, MSC-RC9, GBM-RC9) or HSV-TK (293T-TK, MSC-TK, GBM-TK) genes along with Puromycin resistance gene for selection and Renilla Luciferase (Rluc) gene for detection in cytotoxicity assays (Figure 2a). The dose–response assay showed that all the tested cell types undergo apoptosis very efficiently after exposure to GCV or Rapamycin. In particular, GCV induced cytotoxic activity against 293T-TK cells starting at 10 µg/mL, reaching 78.8% of cell death at 100 µg/mL. Similarly, for MSC-TK, the killing effect was close to 11.9% at 5 µg/mL and 16% at 10 µg/mL of GCV and increased up to 87% at 100 µg/mL. Finally, GBM-TK cells were efficiently eradicated starting at a GCV concentration of 50 µg/mL (40% cell death), reaching 80.3% at 100 µg/mL (Figure 2b). Strikingly, RapaCasp9 demonstrated an even stronger effect with complete eradication of 293T-RC9 and MSC-RC9 cells at a concentration as low as 1 nM (95% and 91% death rate respectively) and elimination of 58.9% of GBM-RC9 cells at that concentration, increasing up to 77.7% at 100 nM (Figure 2c). In all cases, GCV and Rapamycin had no cytotoxic effect on control-transduced cells (Figure 2b ,c), highlighting the specificity of the approach. Consistent with their different mechanism of action, RapaCasp9 showed a fast activation, opposed to TK which took longer time to effectively induce cell apoptosis, suggesting a temporal synergy between the two systems in that one can control cell survival on the short-term while the other on the longer term, if needed. These results show the wide applicability of these suicide systems as single agents on a variety of cell types. In particular, RapaCasp9 which, to date, has not been tested on MSC or tumor cells.

### The double-suicide switch preserves the apoptotic function of both genes in vitro

Next, we engineered different cell types to express a double suicide (DS) system, integrating both RapaCasp9 and HSV-TK along with Puromycin or GFP for selection of transduced cells (293T-DS, MSC-DS, GBM-DS, Figure 2a). The cytotoxicity assay showed that the function of both HSV-TK and RapaCasp9 molecules was preserved and could be activated to induce cell suicide with high efficiency. In detail, apoptosis could be induced in DS cells by incubation with 1 µg/mL GCV, obtaining 23% cell death for 293T-DS, 21% for MSC-DS, and 12.7% for GBM-DS cells. Death rate increased with the dose, reaching 91.2% for 293T DS, 98% for MSC-DS, and 94.2% for GBM-DS at 100 µg/mL (Figure 2d). Moreover, Rapamycin also induced DS cell suicide starting at 0.25 nM, obtaining 68.2% cell death for 293T-DS, 45.3% for MSC-DS and 57.3% for GBM-DS cells. The maximum effect was observed at 100 nM concentration with 69% cell death for MSC-DS, 75% for GBM-DS and 84% for 293T-DS cells. Interestingly, the increase of Rapamycin concentration above 1 nM, increased the cytotoxicity only marginally, showing how a small amount of the inducer molecule is sufficient to obtain the maximum response (Figure 2e). In all cases and conditions, no cytotoxicity was observed in control transduced cells. In conclusion, in vitro experiments show the feasibility of a two-layered suicide system and its versatility in cell lines from different sources.

### The double suicide system effectively controls cell persistence in vivo

**Figure 3.**
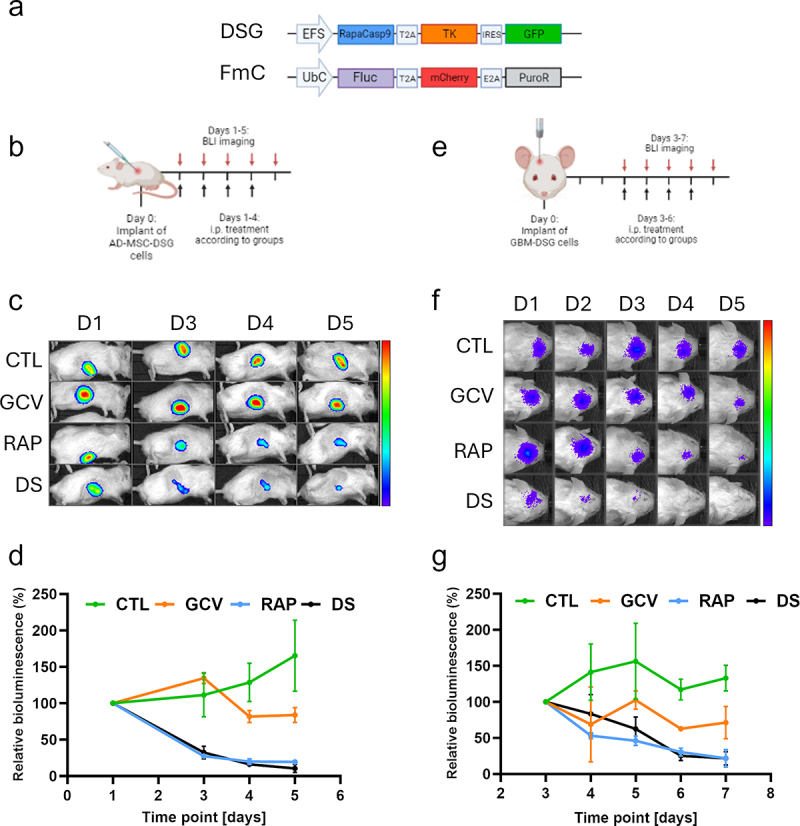
The double suicide kill switch effectively controls transduced cells in vivo. (a) Schematic representation of the constructs. Cells were engineered to express the double suicide cassette along with GFP marker (DSG) and Firefly luciferase along with mCherry and Puromycin resistance (PuroR) for in vivo detection and selection (FmC). (b) Scheme of the in vivo treatment schedule for MSC-DSG model. (c) Representative bioluminescence images showing the change of bioluminescence over time in the different treatment groups of the MSC-DSG model. The same luminosity scale is used for all representative images. (d) Relative viability over time calculated based on the bioluminescence signal for the MSC-DSG model. RAP and DS groups show the greatest reduction in cell viability after 4 days of treatment. GCV demonstrated a low impact on cell survival. Treatment effect was significant by ANOVA (*p* < .01). (e) Scheme of the in vivo treatment schedule for GBM-DSG model. 24 mice were used in this experiment. (f) Representative bioluminescence images showing the change of bioluminescence over time in the different treatment groups of the GBM-DSG model. The same luminosity scale is used for all representative images. (g) Relative viability over time calculated based on the bioluminescence signal for the GBM-DSG model. RAP and DS groups show the greatest reduction in cell viability after 4 days of treatment. GCV demonstrated a lower ability to control the cell growth. Treatment effect was significant by ANOVA (*p* < .05).

To test the efficacy of our double suicide system in vivo, DSG-expressing cells were implanted in NOD.SCID mice and treated with GCV and/or Rapamycin to induce the apoptosis of the cells. In the case of MSC, 4 days of GCV-only treatment was only marginally effective, killing 16.2% of the cells, probably due to its intrinsic characteristic of being better active against fast-growing cells, while MSC have longer cell cycle. On the contrary, Rapamycin alone eliminated 80.6% of the cells, while in Rapamycin + GCV condition, 89.5% of the cells were eradicated, thus overcoming the weakness of TK-only system (Figure 3c, d). In the setting with TCs implanted orthotopically, GCV treatment eliminated 29% of the cells after 4 days of treatment while Rapamycin and Rapamycin + GCV condition killed 78.2% and 78.3% of the cells, respectively (Figure 3d-g). Notably, in both models, administration of Rapamycin resulted in a fast drop in viability, already clear after 1–2 days, while the effect of GCV became evident at a later time point.

### The sequential activation of the suicide switches provides an effective additional safety layer to cell-based therapies

**Figure 4.**
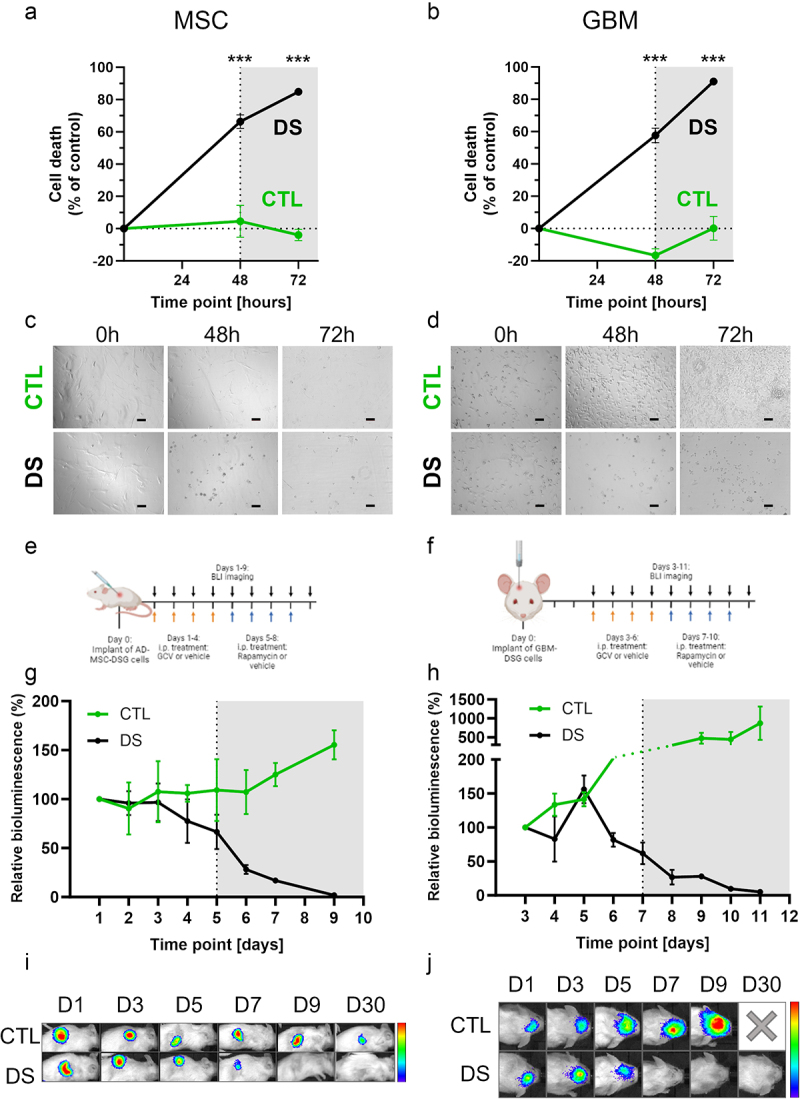
The sequential activation of the kill switches effectively eliminates transduced cells. (a-b) the sequential treatment with GCV for 48 h followed by Rapamycin for 24 h (gray shadow) effectively eliminates MSC DS (a) and GBM DS (b) sparing control cells. (c-d) Microscope photographs of MSC DS (c) or GBM DS (d) and control cells at different time points during the kill switches sequential activation experiment. Scale bars: 100 µm. (e-f) Scheme of the in vivo treatment schedule for MSC-DSG (e) and GBM-DSG (f) models. Black arrows: BLI imaging, orange arrows: GCV treatment, blue arrows: Rapamycin treatment. 20 mice were used in this experiment. (g-h) Relative viability over time calculated based on the bioluminescence signal for the MSC-DSG (g) and GBM-DSG (h) models. In both models, the sequential treatment with GCV followed by Rapamycin (gray shadow) induces a remarkable reduction in cell viability compared to control group. Treatment effect was significant by ANOVA (*p* < .01). (i-j) Representative bioluminescence images showing the change of bioluminescence over time in the treated and control groups of the MSC-DSG (i) and GBM-DSG models (j). For each set of representative images, the same luminosity scale is used.

Next, we treated MSC-DSG and GBM-DSG cells in vitro for 48 h with GCV and subsequently treated them with Rapamycin for another 24 h. GCV treatment induced 66.3% and 57.7% cell death in MSC-DSG and GBM-DSG, respectively. The subsequent treatment with Rapamycin increased the death rate to 84.8% and 91% in MSC-DSG and GBM-DSG, respectively. Control cells underwent the same treatment and showed no toxicity (Figure 4a, b). This efficacy was confirmed in vivo for both cell types. For MSC-DSG, we observed a 33.4% relative cell death after GCV treatment, which further increased up to 98.2% after Rapamycin treatment (Figure 4g). For GBM-DSG cells, we observed a reduction of the signal of 38.2% after GCV treatment, reaching 94.9% after 4 days of Rapamycin administration (Figure 4h). Importantly, mice monitoring up to 30 days did not show any regrowth of either cell types post treatment (Figure 4i, j). In the case of GBM cells, control mice showed severe symptoms associated with tumor growth and were sacrificed at an earlier time.

### The activation of the second switch provides an effective countermeasure in a model of resistance

**Figure 5.**
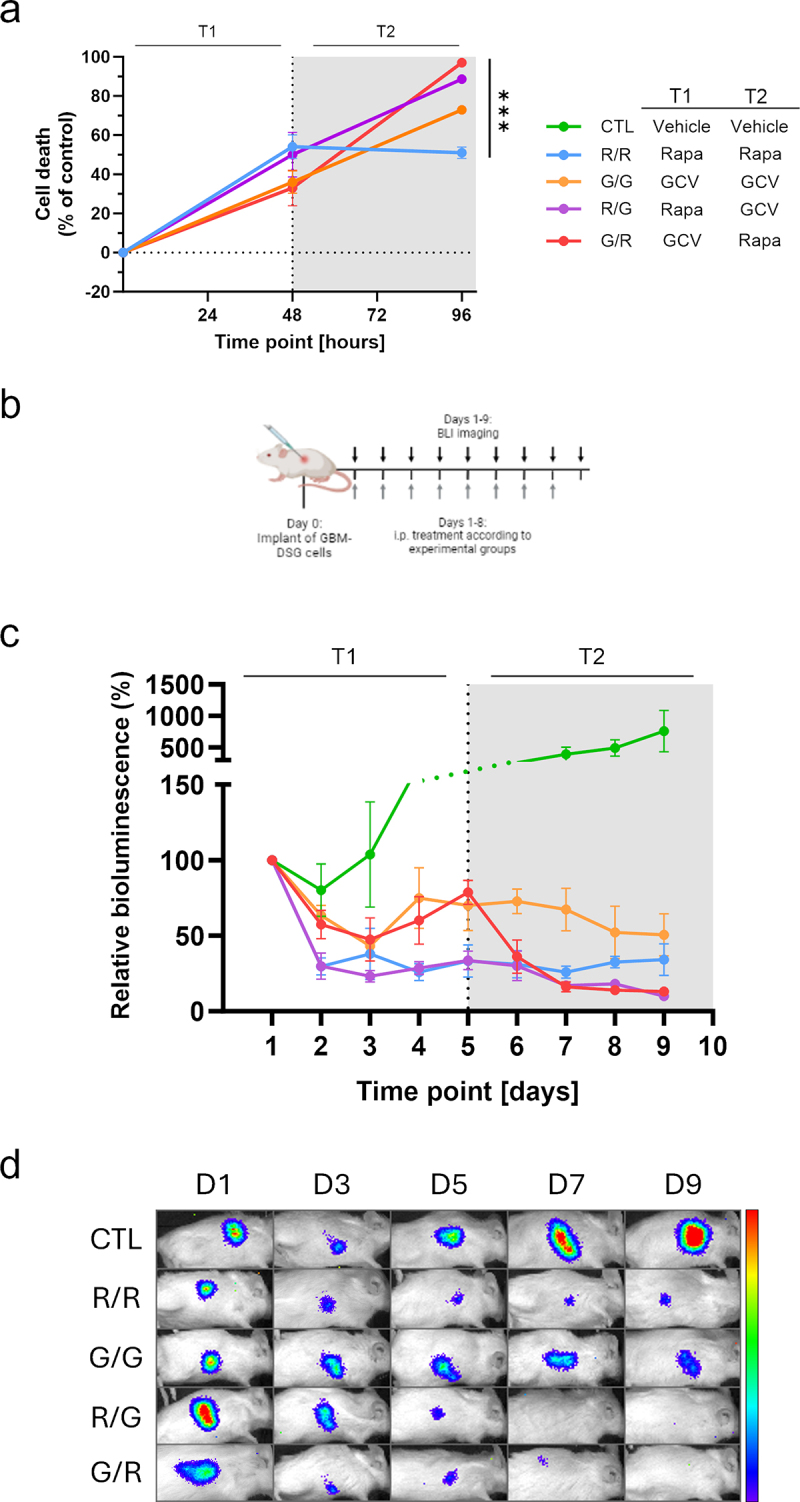
The activation of the double suicide switches eradicates nonsensitive cells in vitro and in vivo. (a) Graph reporting the percentage of cell death in the in vitro model of resistance at 48 h (T1) and 96 h (T2). The treatments for the different experimental groups are summarized in the legend. Sequential treatment with Rapamycin for 48 h followed by GCV for 48 h (R/G group, purple line) or vice versa (G/R group, red line). Prolonged treatments with either GCV only (G/G group, orange line) or Rapamycin only (R/R group, blue line). Cell death between different groups was tested for significance at the last time point using Student’s T Test. ***p < .001. Data for each experimental condition, have been collected from two independent experiments in a technical triplicate. (b) Scheme of the in vivo treatment schedule for the resistance model. Black arrows: BLI imaging, gray arrows: treatment according to the different treatment schedules summarized in the legend above. 20 mice were used in this experiment. (c) Relative viability over time calculated based on the bioluminescence signal for the resistance model in vivo. The experimental groups reflect those of the in vitro assay. Sequential treatment with Rapamycin followed by GCV (R/G group, purple line) or vice versa (G/R group, red line), GCV as a single agent (G/G group, orange line) or Rapamycin as a single agent (R/R group, blue line). Treatment effect was significant by ANOVA (*p* < .01). (d) Representative bioluminescence images showing the change of bioluminescence over time in all the treatment groups. The same luminosity scale is used for all representative images.

We generated a model to reveal the advantages of the double safety switch system in the case of an acquired resistance to the activation of one of the suicide genes. In particular, GBM-DS cells are responsive to both the inducers and represent the sensitive cells while GBM-TK cells are not killed by Rapamycin, thus modeling a resistance to this inducer. In a similar way, GBM-RC9 cells are not killed by GCV treatment and thus can exemplify a resistant population. The three cell types were mixed in equal proportions and tested *in vitro* and *in vivo* in the presence of Rapamycin only, GCV only or both in sequence. *In vitro*, 48 h incubation with Rapamycin, led to 50.1–54.2% of cell death which remained stable (51.1%) when the treatment was prolonged for additional 48 h (Figure 5a, R/R group), consistent with the persistence of a subset of resistant cells. On the contrary, the additional treatment with GCV dramatically increased the death rate to 88.6% (Figure 5a, R/G group) by eliminating the fraction of cells nonsensitive to Rapamycin. In parallel, when cells were treated with GCV for 48 h, a 33–36.1% cell death was observed, which further increased to 72.9% after an additional 48 h of treatment (Figure 5a, G/G group). Almost complete eradication, was achieved only after a Rapamycin treatment, reaching 97.1% cell death at 96 h time point (Figure 5a, G/R group).

In the *in vivo* setting, we observed an outcome consistent with the *in vitro* findings when mice were implanted with the same mixed cell population and treated with Rapamycin only, GCV only or both Rapamycin and GCV in sequence (Figure 5b–d). After 4 days of GCV treatment, the relative bioluminescence signal was 70–78.7%, and decreased to 50.6% when treatment was administered for 4 additional days (Figure 5c, G/G group). Noticeably, when Rapamycin was administered instead, the relative viability dropped to 13.0% (Figure 5c, G/R group), likely due to the eradication of the Rapamycin-only fraction of the mixed cell population which survived to the initial treatment with GCV. In a similar fashion, the treatment with Rapamycin for 8 days led to 34.1% of the cell survival (Figure 5c, R/R group), while 4 days of Rapamycin treatment (33.3% relative viability) followed by 4 days of GCV treatment, reduced the viability to 10% (Figure 5c, R/G group). These results are consistent with the model encompassing a residual population with the ability to escape one layer of safety, but successfully controlled by the activation of both the suicide triggers.

Altogether these results highlight the reliability of the double suicide system, effective on a variety of cells with different biological characteristics, independently of the district of the body they reside. The two-layered system allows a quick elimination of the cells with the additional advantage of a second switch that can be activated later if needed to eradicate cells not responsive to one treatment. The robustness of the approach was confirmed *in vivo* in a model of resistance and by the long-term absence of cell regrowth *in vivo*.

## Discussion

Cell therapies gained a leading role in the innovative treatment of a variety of disorders, including cancer. However, as cell-based therapies are moving rapidly from bench to the bedside, one major concern is their safety with regard to the possibility of uncontrolled proliferation. In this context, suicide genes such as TK and RapaCasp9 among others have been explored as safety switches to eradicate cells in a timely way if needed. In this paper, we propose a two-layered suicide switch, encompassing both the functions of TK and RapaCasp9 in one genetic cassette. Although the single genes have already been employed in different cell-therapy relevant lines, especially immune cells, we here propose the extension and combination of their use. For instance, RapaCasp9 has never been employed on other cells beside immune cells and we here show its efficacy on MSC and GBM cell lines in vitro where it shows a very high efficacy even at a very low concentration of dimerizer. The combined failsafe system that we propose here allows the activation of the second kill switch activation in case the first one is not optimal. Given that different cell types behave differently in in vivo settings, the incorporation of this two-layered system is necessary for cell-based therapies to be translated into human settings. The advantages offered by the system are more relevant in vivo where Rapamycin was able to trigger the cell suicide very effectively, eradicating almost all the cells independently of the cell biological characteristics (fast growing versus slow growing) and even reaching notoriously impervious areas of the organism such as the brain. TK gene, instead, offers a bystander effect that could be useful when dealing with cells growing in a mass, rather than circulating in the bloodstream. A combination of the two genes brings together their advantages and eliminates their weaknesses. In fact, despite being extensively used, HSV-TK is highly dependent on cell proliferation and therefore it is not a perfect system. In our in vivo model, for instance, GCV treatment alone was not sufficient to eradicate the tumor cells although producing a remarkable benefit in comparison to the control group. This could be due to low the intra-tumoral concentration of GCV which was not sufficient to affect the entirety of the solid tumor resulting in surviving cells outgrowing the killing effect. RapaCasp9 gene was able to eliminate the cells that were not controlled by TK alone. Although the simultaneous activation of both genes did not induce a significant improvement in cell eradication efficiency compared to RapaCasp9 only, the model is meant to show the versatility of the combination, introducing a failsafe for the cases where one system alone is not sufficient. In fact, the mechanisms of action of the suicide genes are different and do not overlap, making them an ideal alternate for each other. In particular, we obtained an almost complete eradication of the cells in the model where the two switches were activated in sequence. The strength and translatability of the approach were further explored in a model of resistance where the population of cells was partially unresponsive to either one layer of safety or the other. In this scenario, the sequential activation of both suicide genes successfully eliminated the cells, thus confirming the capability of the two-layered switch to achieve the next level of safety for advancing cellular therapies. For the clinical translation of our approach, the interaction of the suicide switches with other drugs included in patients’ therapeutic regimens should be taken into consideration carefully. In particular, alternatives to GCV, such as Foscarnet, could be used for the treatment of viral infections while many alternatives to Rapamycin are available as an immune suppressant, such as steroids, tyrosine kinase inhibitors, and others.

In summary, we here propose a combination of two suicide genes into one double suicide system, expanding the applicability of the suicide concept to a variety of cell types with different characteristics. We focused in particular on cell types with the potential to grow in vivo, either already clinically relevant such as MSC3 or which translation to the clinical stage is still hindered by safety concerns, such as tumor-derived therapeutic cells^6^. In addition, the incorporation of reliable technology could help move forward those approaches that make use of irradiation as a precaution at the expense of long-term efficacy, such as the case of NK-92-based therapies^17^. The ultimate goal of improving the safety of immune and nonimmune cell-based therapy beyond the current standard is to favor a smoother transition into the clinical setting.

## Materials and methods

### Cell lines and reagents

The human embryonic kidney cell-line 293T and the human GBM cell line U87 (GBM), both from our lab, were cultured in Dulbecco’s Modified Eagle’s Medium (DMEM, Thermo Fisher Scientific Inc.) 4.5 g/L glucose supplemented with 10% Fetal Bovine Serum (FBS, PAA Laboratories Inc., Etobicoke, Canada), 1% penicillin/streptomycin (10,000 U/mL Penicillin, 10 mg/mL Streptomycin in 0.9% NaCl solution, Thermo Fisher Scientific Inc.) and 1% sodium pyruvate (Millipore Sigma). Adipose-derived MSC were purchased from Lonza and cultured in DMEM 1 g/L glucose supplemented with 15% FBS, 1% penicillin/streptomycin, 1% L-Glutamine (Glutamax supplement, Fisher Scientific), 1% Non-Essential Aminoacids (MEM NEAA, Fisher Scientific). Cells were detached when confluent by 0.05% trypsin/EDTA (Life Technologies). All cells were cultured at 37°C in a humidified atmosphere with 5% CO2.

### Vector generation and cell transduction

The DNA encoding RapaCasp9 suicide switch was synthetized based on the sequence provided by Stavrou et al.13. For in vitro assays, the cassettes encoding TK and RapaCasp9 were cloned into third-generation lentiviral plasmid backbones under the control of Ubiquitin promoter and containing Puromycin resistance gene (PuroR) for selection of transduced cells, and Renilla luciferase gene (Rluc) for detection of living cells in the cytotoxicity assays (TK and RC9, Figure 2a). 2A self-cleaving peptides were incorporated at appropriate locations. The double suicide construct (DS) included both the suicide genes along with PuroR in the same backbone as the others (Figure 2a). Control plasmid (CTL) encodes Rluc and PuroR along with mCherry gene as a placeholder (Figure 2a).

For in vivo assays, a modified version of the DS vector was used. In detail, the plasmid included RapaCasp9 and TK genes with GFP as a selection marker, under the control of EFS promoter (DSG plasmid, Figure 3a). The cells were further transduced with a Firefly Luciferase (Fluc) encoding plasmid, to allow in vivo detection of the cells (FmC plasmid, Figure 3a). All the plasmids included a Kozak sequence for a strong initiation of translation and a WPRE at the 3’ UTR to improve mRNA stability.

For virus production, the constructs were transfected into 293T cells according to calcium phosphate-based protocol, along with 3rd-generation helper plasmids. After 48 h, the supernatant containing lentiviral particles was collected and used to transduce 293T, MSCs, and GBM cells. After infection, transduced cells were selected by two consecutive 48 h incubations with Puromycin. DSG-transduced cells were further sorted for GFP expression (FACS Aria III, BD Biosciences).

### In vitro dose-response apoptosis induction assay

Suicide gene-dependent apoptosis induction was tested in vitro in transduced 293T, MSCs, and GBM cells. In detail, for all cell lines, 5000 cells were seeded in the wells of a 96-well black plate and the following day, Ganciclovir (GCV) (Sigma Aldrich) and Rapamycin (MedChem Express) were added to the TK-transduced cells or RC9-transduced cells respectively at different concentrations (1, 5, 10, 50, 100 ug/ml for GCV and 0.25, 1, 5, 25, 100 nM for Rapamycin). After 24 h (for RC9 cells) or 48 h (for TK cells) of incubation, the luminescence emission after incubation with coelenterazine (Caliper Life Sciences) was measured with GloMax-Multi Microplate Reader (Promega Corporation) and cell viability in each condition was estimated as the luminescence signal ratio with respect to untreated control. In case of DS transduced cells, both assays were performed as described and viability was detected by incubating the cells with CellTiter-Glo 2.0 Cell Viability Assay (Promega) before measuring luminescence emission. All experiments were performed two times in technical triplicate.

### In vitro two-layered apoptosis induction assay

The efficacy of the two-layered suicide system was tested in vitro in transduced MSCs, GBM cells and corresponding control cell lines. For all cell lines, 5000 cells were seeded in a 96-well black plate and the following day incubated with 10 µg/mL of GCV for 48 h plus an additional 24 h incubation with 1 nM Rapamycin. Viability was detected by incubating the cells with CellTiter-Glo 2.0 Cell Viability Assay (Promega) before measuring luminescence emission. All experiments were performed two times in technical triplicate. Microphotographs of the cells were taken at several time points in the span of the 72 h treatment window.

### In vitro apoptosis induction assay in a model of resistance

To challenge the two-layered suicide system in the scenario where the cells become resistant to one of the apoptosis inducers, we generated a model by mixing GBM-TK, GBM-RC9, and GBM-DS cells in equal amounts. Mixed cells were seeded in a 96-well black plate and treated according to five different regimens. Rapamycin-only group (R/R) received 1 nM Rapamycin and the treatment was repeated after 48 h. Similarly, GCV-only group (G/G) received 10 µg/mL of GCV, repeated after 48 h. Rapamycin-GCV group (R/G), was incubated with 1 nM Rapamycin for 48 h, followed by 10 µg/mL of GCV for an additional 48 h. Finally, the GCV-Rapamycin group (G/R) was incubated with 10 µg/mL of GCV for 48 h plus an additional 48 h incubation with 1 nM Rapamycin. Control group received no treatment. Viability was detected at 48 h and 96 h by incubating the cells with CellTiter-Glo 2.0 Cell Viability Assay (Promega) before measuring luminescence emission. The cell viability for each group was estimated as the luminescence signal ratio with respect to untreated control. All experiments were performed two times in technical triplicate.

### In vivo double suicide experiment

To test the efficacy of the double suicide system in vivo, two mouse models were prepared. In the first one, 200,000 MSC-DSG-FmC cells in a solution of 50% PBS and 50% Matrigel (Westnet) were subcutaneously implanted in the flank of 12 NOD.SCID mice. The following day, in vivo luminescence signal was measured using IVIS Lumina system (Perkin Elmer) and the mice were randomly subdivided into four groups according to different treatment schedules. Control group (CTL) received 100 µL of vehicle (6.25% DMSO in PBS) i.p.; Ganciclovir only group (GCV) received 50 mg/Kg of GCV i.p.; Rapamycin only group (RAP) received 5 mg/Kg of Rapamycin i.p.; Double suicide group (DS) received both 50 mg/Kg of GCV and 5 mg/Kg of Rapamycin i.p. Mice were treated and imaged every day for 4 days. A final bioluminescence image was taken at the 5th day after cell implant (Figure 3b).

For GBM model, 50,000 GBM-DSG-FmC cells were stereotactically implanted in the right cerebral hemisphere of 12 NOD.SCID mice (2 mm lateral from bregma, 1.5 mm dorsal and 2.5 mm ventral). Three days after implant, the mice were divided into four groups and treated as described above for MSC cells (Figure 3e).

At the end of the study, the luminescence signal for every mouse was normalized with the signal recorded at the first time point and used as a measure of relative cell viability. For the duration of the experiments, mice were housed at 12 h light, 12 h dark cycle with no restrictions on food and water supply. All in vivo procedures were approved by the Subcommittee on Research Animal Care at Brigham and Women’s Hospital. Animals were randomly allocated to cages and experimental groups.

### In vivo two-layered suicide model

To test the approach in a more case-specific scenario, two mouse models were generated. MSC-DSG-FmC and GBM-DSG-FmC cells were implanted in NOD.SCID mice as described above (*N* = 10 mice each). For each model, mice were randomly assigned to two groups. Control group (CTL) received 100 µL of vehicle (6.25% DMSO in PBS) i.p. for 8 consecutive days; Double suicide group (DS) received 50 mg/Kg of GCV i.p. for 4 days followed by 5 mg/Kg of Rapamycin i.p. for 4 additional days. From the first day of treatment, mice were imaged every day for 9 days, plus an additional image at 30 days post-implant. At the end of the study, the luminescence signal for every mouse was normalized with the signal recorded at the first time point and used as a measure of relative cell viability.

### In vivo two-layered suicide in a model of resistance

To further validate the two-layered safety switch, we tested the approach in a model where a subpopulation of cells becomes resistant to one of the apoptosis inducers. In particular, GBM-DSG-FmC cells were mixed in equal amount with GBM-TK-FmC and GBM-RC9-FmC cells in a solution of 50% PBS and 50% Matrigel (Westnet) and subcutaneously implanted in the flank of 20 NOD.SCID mice (60,000 cells total). The following day, in vivo luminescence signal was measured using IVIS Lumina system (Perkin Elmer) and mice were randomly subdivided into five groups according to different treatment schedules. Control group (CTL) received 100 µL of vehicle (6.25% DMSO in PBS) i.p. daily for 8 days; Ganciclovir only group (G/G) received 50 mg/Kg of GCV i.p. daily for 8 days; Rapamycin only group (R/R) received 5 mg/Kg of Rapamycin i.p. daily for 8 days; Rapamycin-GCV group (R/G) received 5 mg/Kg of Rapamycin i.p. daily for 4 days, followed by 50 mg/Kg of GCV i.p. daily for 4 more days; GCV-Rapamycin group (G/R) received 50 mg/Kg of GCV i.p. daily for 4 days, followed by 5 mg/Kg of Rapamycin i.p. daily for 4 more days. From the first day of treatment, mice were imaged every day for 9 days. At the end of the study, the luminescence signal for every mouse was normalized with the signal recorded at the first time point and used as a measure of relative cell viability.

### Statistical analysis

Pairwise statistical analyses were performed using Student’s T test while time course experiments were analyzed with ANOVA (GraphPad PRISM ver. 9.3.1). A p-value less than or equal to 0.05 was considered significant.

## Data Availability

The data that support the findings of this study are available from the corresponding author, K.S., upon reasonable request.
