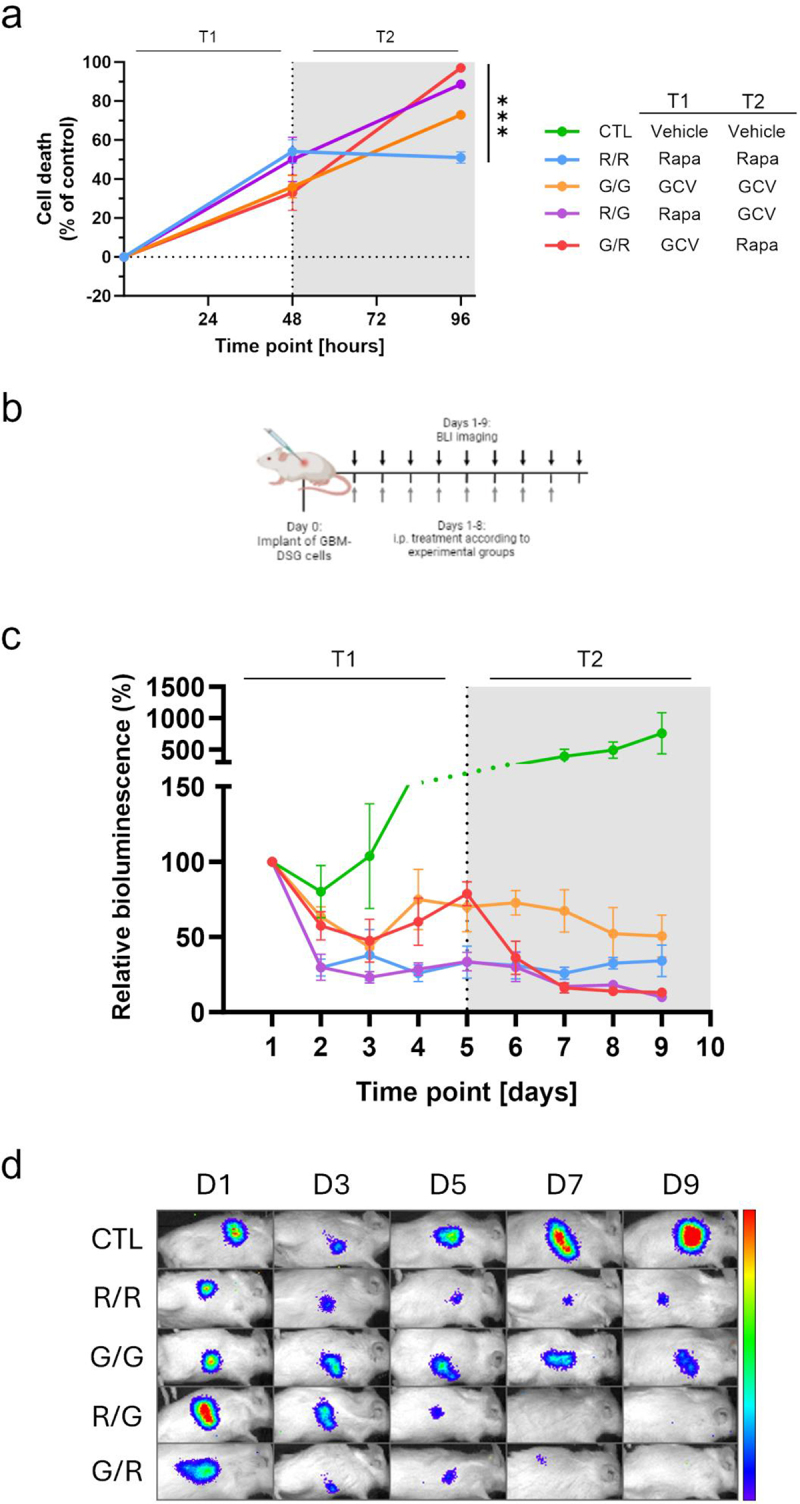# Correction

**DOI:** 10.1080/15384047.2024.2392996

**Published:** 2024-08-19

**Article title**: Developing and characterizing a two-layered safety switch for cell therapies

**Authors**: Filippo Rossignoli, Danielle Hoffman, Emaan Atif, and Khalid Shah

**Journal**: Cancer Biology & Therapy

**DOI**: https://doi.org/10.1080/15384047.2023.2232146

Authors have been notified of an image duplication that exists in the panels depicted in Figure 3f and Figure 5c. For Figure 3f, this was an inadvertent error in placing representative images for the in vivo BLI plots shown in Figure 3g. Similarly, Figure 5d is intended to provide representative images for the in vivo BLI plots shown in Figure 5c. The correction for both these figures has now been made. Because the images are merely representative of the data, these corrections have no impact on the conclusions expressed in Figure 3 and 5 or elsewhere in the paper. The authors would like to thank the journal for an opportunity to clarify and correct these points and apologize for any inconvenience caused.

**Figure 3:**
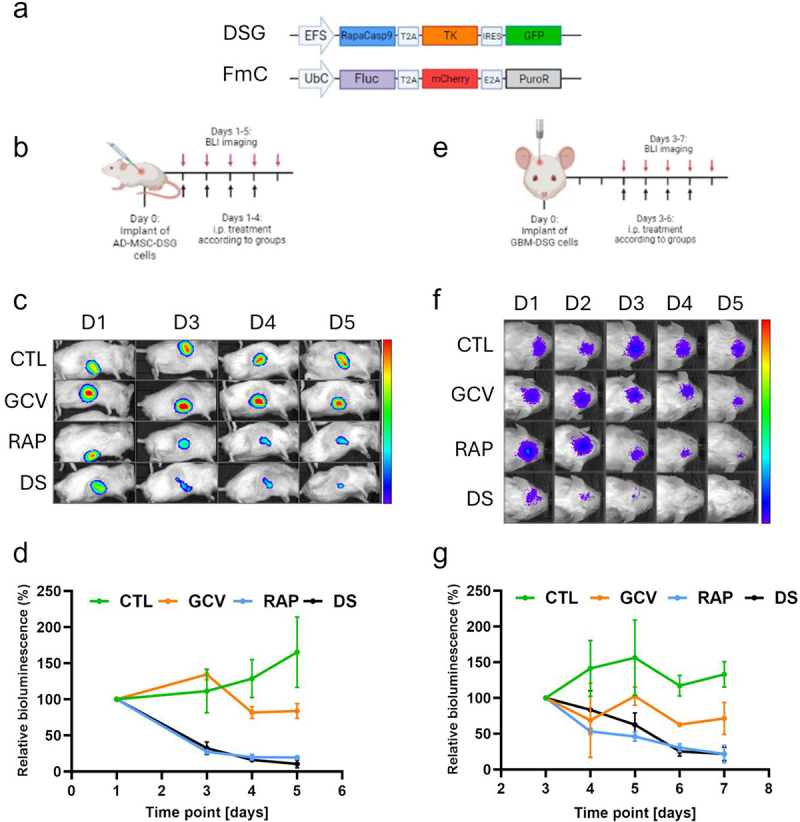

**Figure 5:**